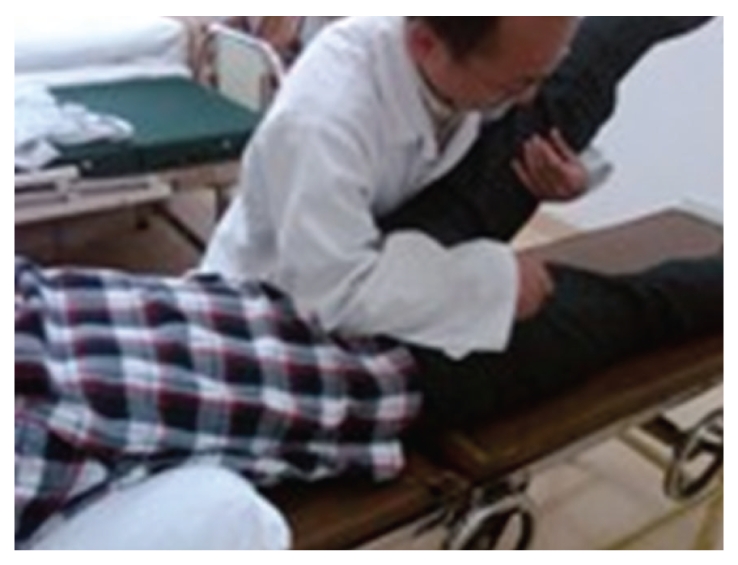# Hematuria after Orthopedic Tuina: A Case Report and Brief Review of Literature

**DOI:** 10.1155/2011/953686

**Published:** 2011-03-15

**Authors:** Xiao-Ming Ying, Peng Wang, Ben-Shun Yao, Hai-Yang Gu, Quan-Zhen Xu

**Affiliations:** Department of Tuina, The Third Hospital Affiliated Zhejiang University of Traditional Chinese Medicine, 219 Mogangshang Road, Hangzhou 310004, China

## Abstract

We present a case of a 24-year-old man who presented hematuria after the orthopedic tuina, which has not been recorded previously in the literature. We review complications of tuina in the literature too.

## 1. Introduction

Tuina is an important part of Chinese medicine, which has been a therapeutic method for several thousand years in China. Orthopedic tuina is a branch of Chinese traditional manipulation. Eight traumatic and orthopedic manipulations were recorded in *Golden Mirror of Medicine*, which included* mo*, *jie*,* duan*,* ti*,* an*,* mo*,* tui*, and* na* in Qing dynasty. Although orthopedic tuina is an important therapeutic method to spinal disease in China, the complications of tuina are seldom reported in the literature. 

The paper describes a patient with lumbar disc herniation, who experienced hematuria after orthopedic tuina. In order to learn the adverse effects of tuina, a comprehensive literature search was performed on Medline, Embase, and CNKI (China), with *adverse events*,* complicatiion*,* safety*,* tuina*,* and massage* as search terms*.*

## 2. Case Report

**Figure 1:**
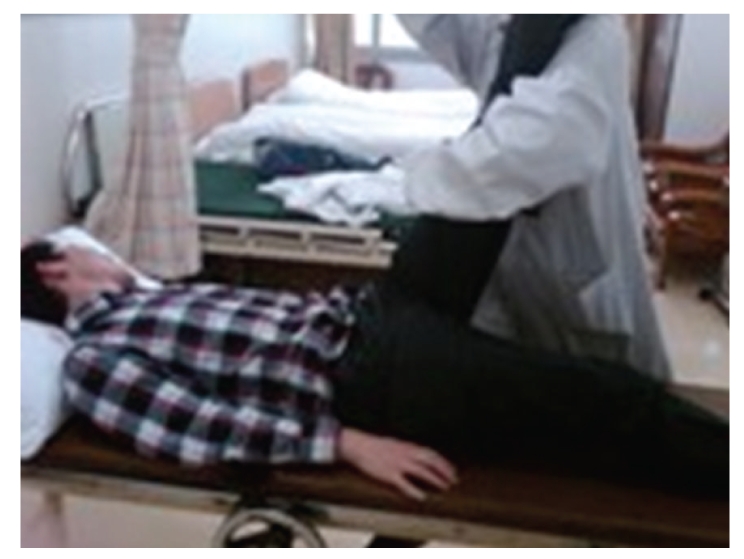


**Figure 2:**
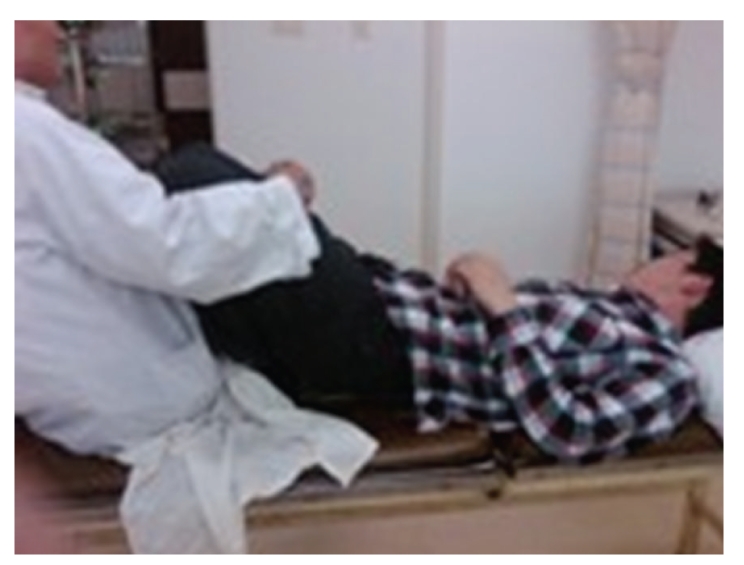


**Figure 3:**
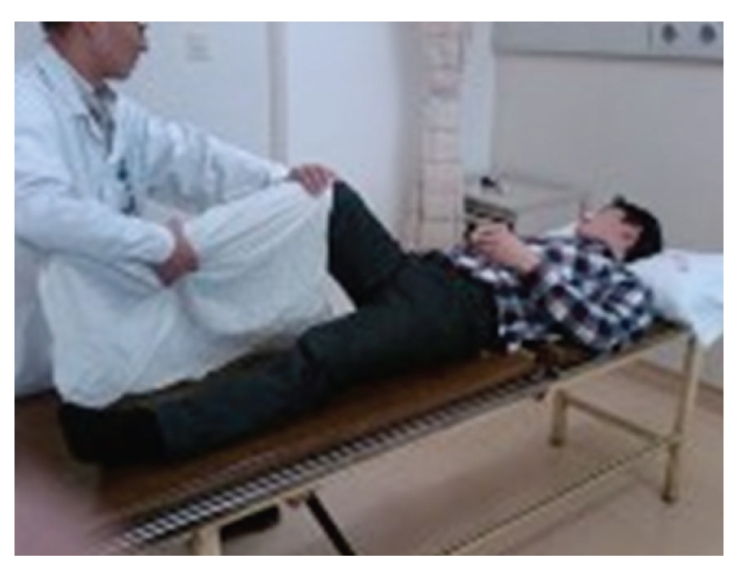


 A 24-year-old Chinese male was admitted to our hospital in March 2009 for lumbar disc herniation. After the elimination of the contraindication, orthopedic tuina was conducted. Process followed: (1) pressed left or right foot in turn with straight leg raising 50 times each (Figure 1), (2) shaked low back with bending hip and knee 100 times (Figure 2), (3) drew left and right lower limbs in turn 3 times each (Figure 3), and (4) On prone position, pulled manipulation on low back with extension of lower limbs twice (Figure 4). The treatment was successful, and there was no uncomfortableness in the process. The patient was instructed to rest in bed. On the first day, 300 ml brown urine appeared after he turned round. Urine-Rt urgently: occult blood 2+, protein 2+, microscopic examination: RBC 1+. We used aminomethylbenzoic acid to stop bleeding. On the second day, patient presented hematuria again, and three-glass test showed hematuria in the whole process of urination; red blood cell morphology examination notified mass red blood cell, 5 percent of RBC was swollen and central olistherozone swelled; 65 percent of RBC had burr. Abnormal sign was not found on ultrasound and computer tomography of kidneys. Contusion of kidney was considered, and the patient was instructed to rest in bed and orally took aminomethylbenzoic acid continually. Hematuria disappeared ten days later. 

## 3. Discussion

Tuina is a physical therapy, and the adverse effect or complications are seldom. Hematuria and contusion of kidney were not mentioned in any literatures searched. Abnormal occurrence of tuina has broken skin, bleeding and seldom syncope, fractures [1]. Hematoma was the common complication of tuina therapy [2–4]. A 66-year-old male presented the swelling of the neck and arms, which limited his daily activities after 10 cycles of infrared heat and massage therapy approximately 1 month [5]. A patient with tennis elbow presented posterior interosseous nerve palsy after friction massage [6]. The severe complications reported include the rupture of internal organs such as uterus and colon [7, 8]. A 72-yr-old woman with leg pain presented pulmonary embolism after massaging calf muscle [9]. In addition, a 59-yr-old man with aortofemoral bypass suffered from the embolization of the left kidney when the back massage including walking on back was conducted [10]. A 57-yr-old woman with Hashimoto's disease experienced transient destructive thyrotoxicosis because of massaging neck likely [11]. A 38-yr-old diabetic man with peripheral neuropathy was treated by vacuum boot foot massage with mechanical device, who suffered from ulceration and infection and had to amputate the leg lastly [12]. Unfortunately, a 56-yr-old woman was dead due to the strangulation of neck when she rolled the neck herself with electrical roller massage device [13]. A 16-yr-old boy with exostosis on femur treated by traditional Chinese massage on the right thigh because of pain of the right thigh, presented pseudoaneurysm of popliteal artery [14]. Kerr HD reported that a 51-yr-old woman with ureteral stent experienced displacement of ureteral stent after her abdomen, pelvis, and lower back experienced deep body massage using Rolfing technique [15]. 

In China, a patient with lumbar disc herniation suffered from deep vein embolism of left lower extremity when she was treated with deep manipulation [16]. Among 600 patients who were massaged by being hung upside down, 87 cases experienced complications, including syncope, pain, and getting tired [17]. The patient in the present paper experienced hematuria after orthopedic tuina. We think that the patient's kidney was injured when he was pulled low back with extension of lower limbs because the patient was thin, point of action of pulling method was uncertain, and power was heavy; range of technique was too wide. 

In summary, there are various complications of tuina despite of lower incidence, and a few complications are severe, even destructive. The masseur should avoid complications with various methods; for instance, choosing the different power, range, and techniques according to condition of patients. 

## Figures and Tables

**Figure 4:**